# The erosive effect of pomegranate juice on enamel: An in vitro study

**DOI:** 10.1371/journal.pone.0298404

**Published:** 2024-04-10

**Authors:** Yue Chen, Zeyu Wu, Peng Sun, Jie Song, Yishan Liu, Jin Zhao

**Affiliations:** 1 Department of Pediatric Dentistry, The First Affiliated Hospital of Xinjiang Medical University (The Affiliated Stomatology Hospital of Xinjiang Medical University), Urumqi, China; 2 Stomatology Disease Institute of Xinjiang Uyghur Autonomous Region, Xinjiang Medical University, Urumqi, China; 3 Department of Cariology and Endodontics, The First Affiliated Hospital of Xinjiang Medical University (The Affiliated Stomatology Hospital of Xinjiang Medical University), Urumqi, China; 4 Xinjiang Institute for Drug Control, Urumqi, China; University of Puthisastra, CAMBODIA

## Abstract

**Aim:**

Dental erosion is a chemical-mechanical process that leads to the loss of dental hard tissues. This study aimed to investigate the effect of pomegranate juice on the enamel.

**Methods:**

Enamel blocks were randomly divided into three groups: deionized water, cola, and pomegranate juice. The blocks were immersed in the solutions four times a day for 14 days, and stored in artificial saliva for the remaining period. The surface hardness was measured on days 7 and 14. The surface structures of the demineralized blocks were observed via scanning electron microscopy (SEM), and the depth of demineralization was observed via confocal laser scanning microscopy (CLSM). The pH, calcium, and phosphorus levels of the three solutions were analyzed.

**Results:**

The microhardness values of the blocks in the pomegranate juice and cola groups decreased with the increase in the demineralization time. The blocks in the pomegranate juice group exhibited large fractures in the enamel column, whereas those in the cola group had pitted enamels with destruction of the interstitial enamel column. Compared with cola group, fluorescent penetration increased in pomegranate juice (*P* < 0.01). The pH of cola (2.32 ± 0.09) was lower than that of pomegranate juice (3.16 ± 0.16). Furthermore, the calcium content in pomegranate juice was significantly higher than that in cola (*P* < 0.01). Alternatively, the concentration of phosphorous in cola was significantly higher than that in pomegranate juice (*P* < 0.01).

**Conclusion:**

These findings indicate that pomegranate juice can cause enamel demineralization with an erosive potential comparable to that of cola.

## Introduction

Dental erosion (DE) refers to an oral disease characterized by the progressive and irreversible loss of dental hard tissue caused by the action of acid mist or anhydride; the demineralization process is acid-related and not bacteria-related. The prevalence of dental erosion ranges between 30% and 50% in deciduous and 20% and 45% in permanent teeth [1]. Dental erosion is a chemical-mechanical process that leads to persistent loss of dental hard tissue. It can affect any tooth surface and cause complications, such as enamel loss, reduced aesthetics, and tooth sensitivity [2]. Acidic beverages lower the pH around the enamel, destroying the hydroxyapatite structure, causing loss of enamel surface structure, and triggering irreversible loss of enamel surface material [3]. Therefore, it is crucial to understand the acid-etching potential of various beverages on the enamel and form a theoretical basis for preventing tooth erosion.

Pomegranate (*Punica granatum L*.) is a medicinal and edible plant resource with abundant nutrients and various health benefits [4]. The solubility, solid content, grain color saturation, malic acid content, total anthocyanin content, and total phenol content of the pomegranate in Xinjiang Kashgar are higher than those produced in the other areas [5]. Pomegranate is rich in dietary fiber and health nutrients, including vitamins (vitamins C, A, and folic acid), minerals (potassium, magnesium, and copper), phenolic compounds, alkaloids, triterpenes, and sterols [6]. Furthermore, several studies have demonstrated the role of pomegranate in reducing the risk of cardiovascular disease, diabetes, cancer, and Alzheimer’s disease [7–11].

There is a growing interest in the effects of fruit juice on teeth owing to the increase in its consumption in recent years [12]. According to Barbour and Lussi, it is complicated, but not impossible, to rank the acid erosion potential of different acidic beverages [13]. In recent years, studies have confirmed the beneficial effects of pomegranate juice in preserving dislocated teeth and inhibiting the number of colonies formed by Streptococcus and Lactobacillus [14]. However, studies investigating the effect of pomegranate juice on enamel demineralization are lacking. Therefore, the aim of the present study was to measure the surface hardness on days 7 and 14 using a Micro Vickers Hardness Tester with cola as a positive control and deionized water as a negative control. The surface structure of the demineralized samples was observed using scanning electron microscopy (SEM) and the depth of demineralization was observed using laser confocal microscopy (CLSM). The pH, calcium concentration and phosphorus concentration of pomegranate juice, cola and deionized water were analyzed to assess the demineralization capacity of pomegranate juice and to provide a theoretical basis for the prevention of dental erosion.

## Materials and methods

### Sample preparation

A sample size of eight was initially calculated based on the study by Santos et al. [15]. Subsequently, the size was increased to 10 in each experimental group to compensate for possible loss during the experiment [15]. In October-November 2021, 30 freshly extracted maxillary incisors from cattle belonging to the same age range were obtained from the Urumqi Xishan slaughterhouse (Urumqi, Xinjiang, China) and preserved in 0.1% bromothymol solution (Beijing Solarbio Technology Co., Ltd., Item No. T8690). The residual soft tissues of the crowns were removed using tissue scissors. After removing the piths, the crowns were rinsed under deionized water (Beijing Solarbio Technology Co., Ltd., Item No. F0025) and set aside. Bovine incisors with well-developed enamel, no caries, no pigmentation, and no apparent cracks were selected using a stereomicroscope. The crowns and roots were separated under running water using a grinding machine (KaVo Group-Shanghai Headquarters, China, 901). Enamel blocks (5 × 5 × 2 mm) were prepared from the crowns of the bovine incisors, and the edges were smoothed with a grinding stone. The surface of the block was smoothened and polished by removing about 150 μm of the enamel surface with silicon carbide sandpaper (800, 1200, and 2400 mesh) under the deionized water. The 30 prepared enamel blocks were placed in an ultrasonic cleaner and swished for 8 min to remove the dirt. A horizontal rectangular area (4 × 4 mm) was drawn on the middle of each enamel surface with a carbon pencil and was used as the opening on the surface; the remaining areas were covered evenly with a double layer of nail polish (Jinhua Mengni Cosmetics Co., Ltd.). The blocks were washed with deionized water and placed in 0.1% bromothymol solution until use.

### Experimental grouping and treatment

Thirty enamel blocks with initial hardness values ranging from 310 to 390 HV were selected and grouped using the random number method. The blocks were divided into three groups based on the solution as follows (n = 10 each): deionized water, cola (Coca-Cola Beverages Xinjiang Co., Ltd.), and pomegranate juice (Xinjiang Ruitai Fruit Development Co., Ltd.). The blocks were placed in 15 mL centrifuge tubes containing 10 mL of deionized water, cola, or pomegranate juice and demineralized for 90 s four times a day (10:00, 12:00 noon, 14:00, and 16:00) for a total of 14 days. The blocks were placed in a thermostat and maintained at 37°C during demineralization. After demineralization, the blocks were rinsed for 15 s using deionized water and immersed in artificial saliva (pH, 6.8; Beijing Solarbio Technology Co., Ltd., Item No. A7990) for the remaining period until the next session. The artificial saliva consisted of deionized water, NaCl, KCL, Na_2_SO_4_, NH_4_Cl, CaCl_2_ · 2H_2_O, NaH_2_PO_4_ · 2H_2_O, CN_2_H_4_ONaF. The treatment solutions (deionized water, cola, and pomegranate juice) were collected each day and stored at -80°C.

### Microhardness measurement

The surface hardness of the block was measured using a fully automatic Micro Vickers Hardness Tester (Model: ZHV μ-A; Zwick [China] Co.). The enamel block was placed on the stage with the fenestration facing upwards, and the measurement range was determined at 10×. A diamond prismatic indenter was used to apply a force of 200 g for 10 s. The area of the rhomboid pattern indented on the enamel fenestration surface was calculated by measuring the enamel surface microhardness value at 40×, and the surface microhardness of the fenestration area of the enamel block was determined using a formula on the computer settings. Five points (each 100 μm apart) were measured on the fenestrated surface of each block, and the hardness values of the five points were calculated; the mean value was considered as the mean hardness value of each enamel block. Images of the blocks were obtained at 40×. The hardness value measured before demineralization (initial hardness value) was denoted as SMH1. On the 7^th^ and 14^th^ day of demineralization, the hardness values were determined using the same method and recorded as SMH2 and SMH3, respectively.

### Scanning electron microscopy (SEM) observation

SEM (Nippon Electron Co., Ltd., Model: JSM-6390LV) was used to evaluate the morphological changes on the enamel surface after acid etching. One block was randomly selected from each group on day 14 and fixed with 2.5% glutaraldehyde for 1 h, followed by washing with 0.1M phosphate buffer (pH, 7.2) three times for 20 min each. After gradient dehydration with 50%, 70%, 80%, 90%, and 100% tert-butanol (15 min each), the blocks were washed with deionized water for 15 min. The blocks were then dried in a JEOLJFD-320 cold ice drier, fixed on the carrier table with the glazed open side up, and sprayed with gold under vacuum. The surface morphology of the enamel opening surface on the block was observed under SEMs. The blocks were observed under low magnification followed by high magnification to examine the surface morphology of the enamel in the experimental area of the block, and images were recorded at 3000×, 6000×, and 10,000×, respectively.

### Staining and confocal laser scanning microscopy (CLSM)

On day 14, one randomly selected demineralized block per group was sectioned into 3–5 slices (thickness, 300 μm) using a Kavo sander under running water. The prepared enamel slices were placed in rhodamine B solution (Sigma-Aldrich (Shanghai) Trading Co., Ltd., Item No. 02558) under protection from light, and placed in a constant temperature incubator at 37°C for 48 h for staining. Subsequently, deionized water was used to rinse the blocks three times (5 min each) to wash away the dye on the surface. The blocks were dried and placed on slides, sealed with glycerol, and viewed under a laser scanning confocal microscope (Leica Measuring Instruments (China) Co., Ltd., Model: TCS-SP8) to observe the fluorescence penetration in the demineralized areas. Image Pro Plus 6.0 (Media Cybernetics, Rockville, MD, USA) software was used to analyze the enamel fluorescence area (Area, μm^2^), total fluorescence in the measured area (IOD), and mean fluorescence in the measured area (MIOD) values using the following formula: MIOD = IOD/Area

### pH measurement

The baseline pH of all beverages was measured at two time points: immediately after opening the package and after 24 h. The measurements were taken after 24 h because, in some instances, the beverages were not wholly consumed immediately after preparation. The test solution was shaken before each measurement to mix the components thoroughly. The pH value of each experimental solution was measured using a pH meter (Shanghai Lei Ci Instrument Factory, model PHS-3B). The measurement was repeated three times for each group, and the average was taken as the final pH value of the solution. The pH meter was calibrated before each use.

### Calcium measurement

On the 14^th^ day of demineralization, the calcium ion concentration was measured via copper complexation with o-cresyl peptide. The test solution and calcium phosphate ion kit (Shanghai Biyuntian Biotechnology Co., Ltd., Cat. No.: S1063S) were removed from the refrigerator and placed at room temperature. Incubate for 5–10 min at room temperature away from light, measure the optical density value at 575 nm by an enzyme standardizer (ThermoFisher, model Multiskan GO) and generate a standard curve. The calcium content in the block was determined by calculating the average absorbance of each concentration group in the standard and subtracting the absorbance of the blank control group. The standard curve was plotted using the amount of calcium ions in the calcium standard as the horizontal coordinate and the absorbance as the vertical coordinate. The calcium content in the different solutions was calculated from the standard curve.

### Phosphorus measurement

The phosphorus ion concentration was detected using the phosphomolybdic acid method (Beijing Solarbao Technology Co., Ltd.; item no. BC2850). Concentrated sulfuric acid (1.0 mL) was added to 0.1 mL of the solution to be examined in a test tube and placed in a boiling water bath for 10 min. After cooling the mix to room temperature, 200 μL of the reagent was added and mixed; the mix was then placed in the boiling water bath until a transparent solution was observed. The tube was then removed from the bath and cooled to room temperature, followed by the addition of 3.8 mL of distilled water. The mix was centrifuged (Model: 3-18KS; Sigma-Aldrich [Shanghai] Trading Co., Ltd.) at 10000 rpm for 10 min, and the supernatant was collected for measurement. The phosphorus ion concentration was calculated based on the concentration of the standard solution.

### Statistical analysis

**Fig 1:**
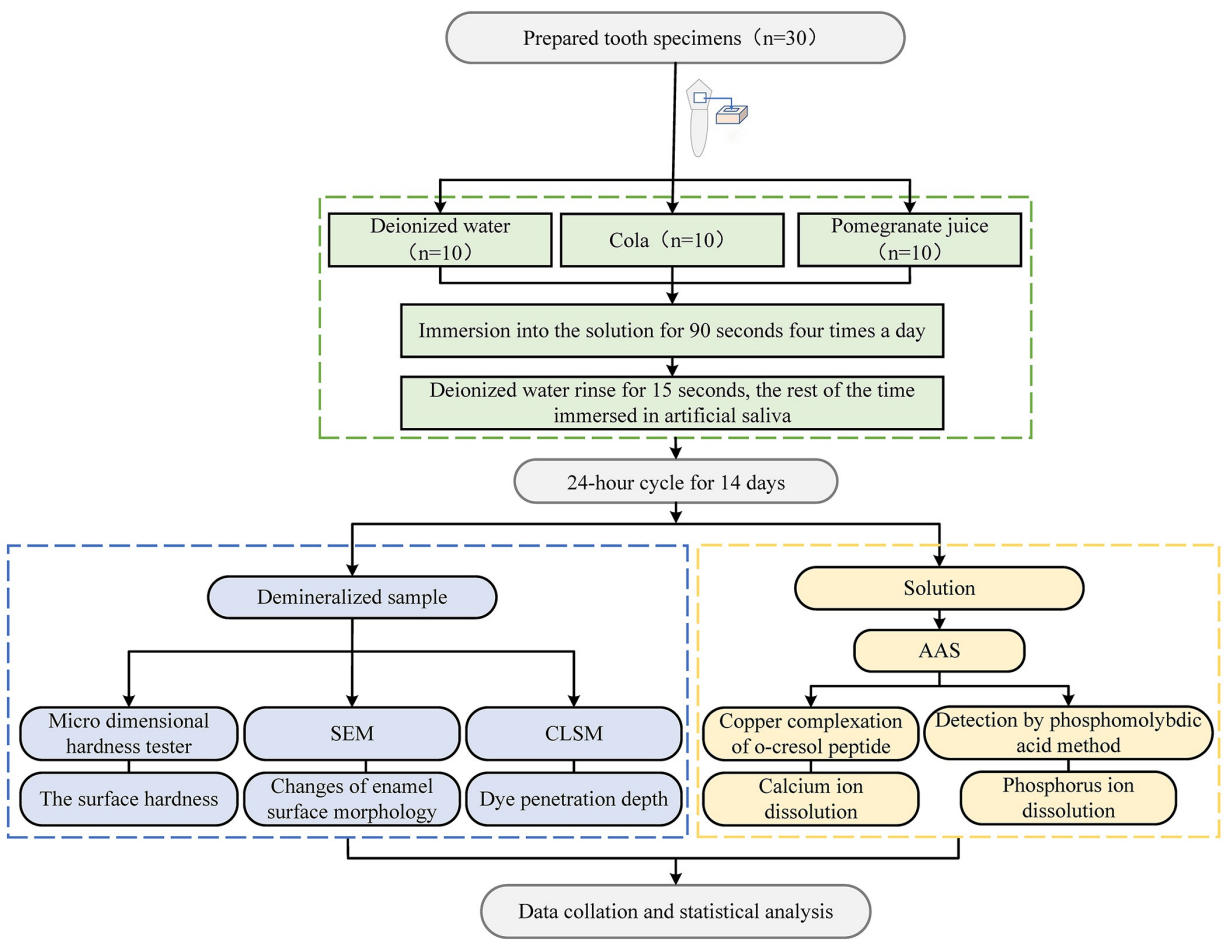
The experimental flowchart.

SPSS statistical software (SPSS 26.0: SPSS; Chicago, IL, USA) was used to analyze the data, which conformed to the normal distribution and are expressed as the mean ± standard deviation ($\bar{x}$ ± s). The data from each group were tested for homogeneity of variance using the LSD two-sided test. The Kruskal–Wallis rank sum test was performed to test the heterogeneity of variance, with a test level of α = 0.05. According to Li et al. ’s research experimental method [16], we modified it and plotted the experimental flowchart, as shown in Fig 1.

## Results

### Surface morphology

The enamel surface was tarnished, and pigmentation was observed in specimens from the cola and pomegranate juice groups. In the cola group, the tooth surface turned dark brown, and the color gradually darkened from the center to the periphery. Alternatively, the color of the tooth surface in the pomegranate juice group was tan and uniformly distributed. The tooth surface of the blocks in the deionized water group appeared chalky.

### Surface microhardness values

**Fig 2:**
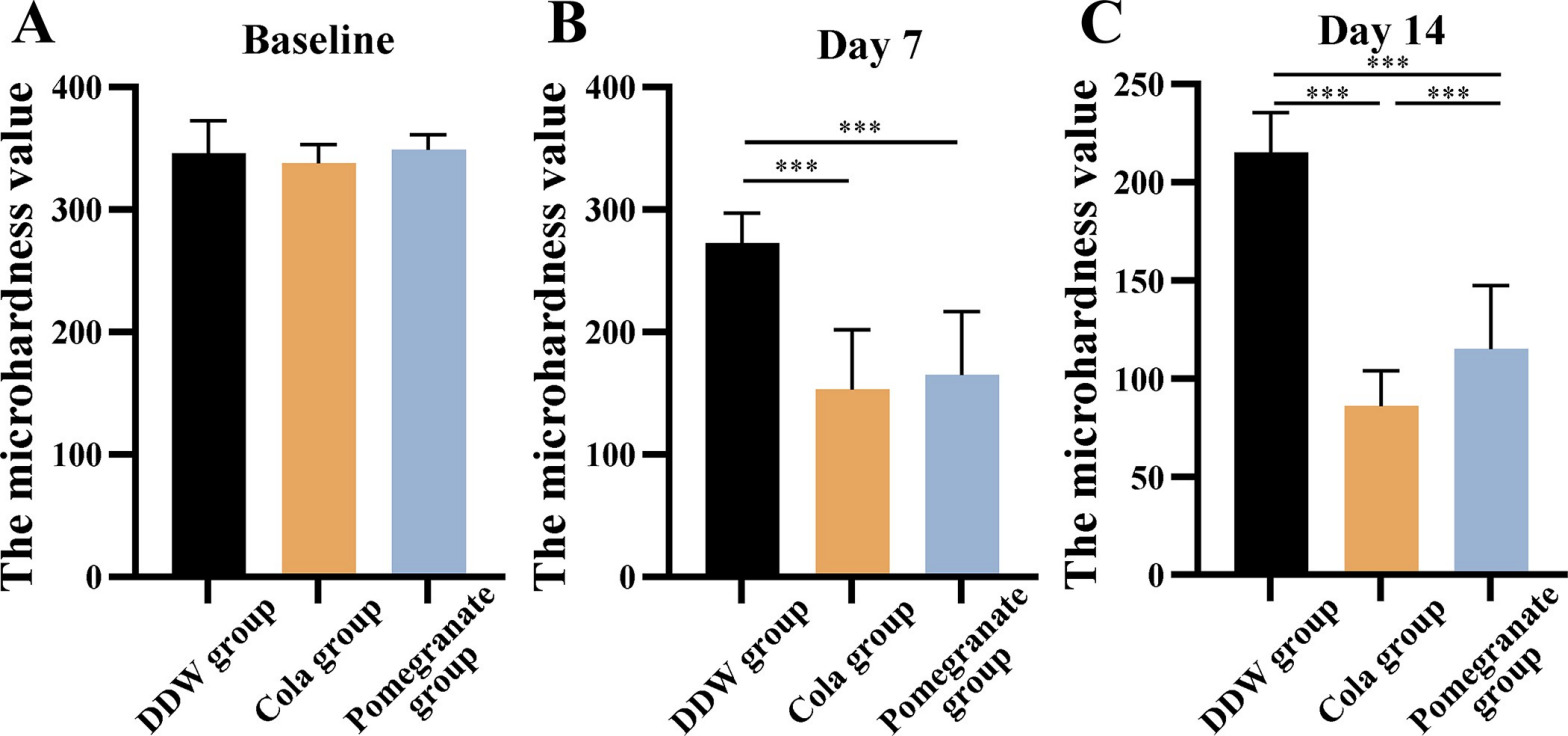
Comparison of the surface microhardness values of the specimens between the control and experimental groups at each time point (n = 10, mean ± SD). (A) Initial microhardness value. (B) Microhardness values at day 7. (C) Microhardness values at day 14. *P < 0.05, **P < 0.01, ***P < 0.001.

As shown in Fig 2A, the baseline surface microhardness values in the groups were similar, with no statistically significant differences. However, statistically significant differences were observed between the pomegranate juice and cola groups and the deionized water group on days 7 and 14 after demineralization (Fig 2B). On day 14 of demineralization, the microhardness value in the pomegranate group was significantly lower than that in the cola group, indicating that the pomegranate juice had a stronger demineralizing propensity than cola (Fig 2C).

### SEM

**Fig 3:**
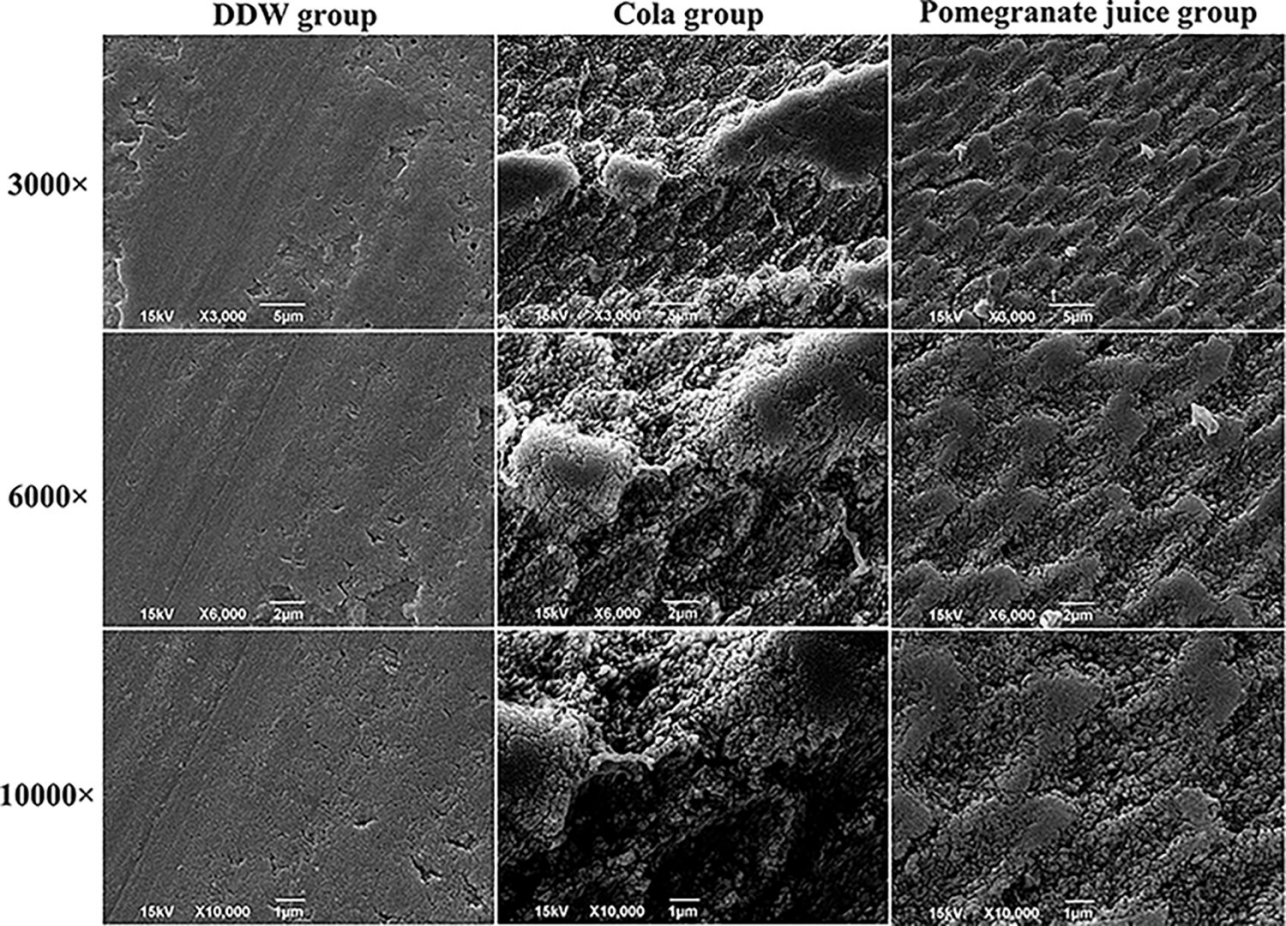
SEM images of the surfaces of specimens from the deionized water, cola, and pomegranate juice groups.

The surface morphology of the blocks in the deionized water group was essentially intact and homogeneous, as observed under low magnification; only small scattered depressions were seen on the surface under high magnification. Dense, rough pit-like structures were seen on the enamel surface in the cola group, with interstitial destruction of the enamel columns in the form of fissures and partially dissolved and ablated areas connected in sheets. The enamel surface of the specimens in the pomegranate juice group was rough with many pores. Additionally, large fractures in the enamel columns, dissolution of the center of the columns, and a honeycomb structure with a concave surface connected in sheets were observed (Fig 3).

### CLSM

**Fig 4:**
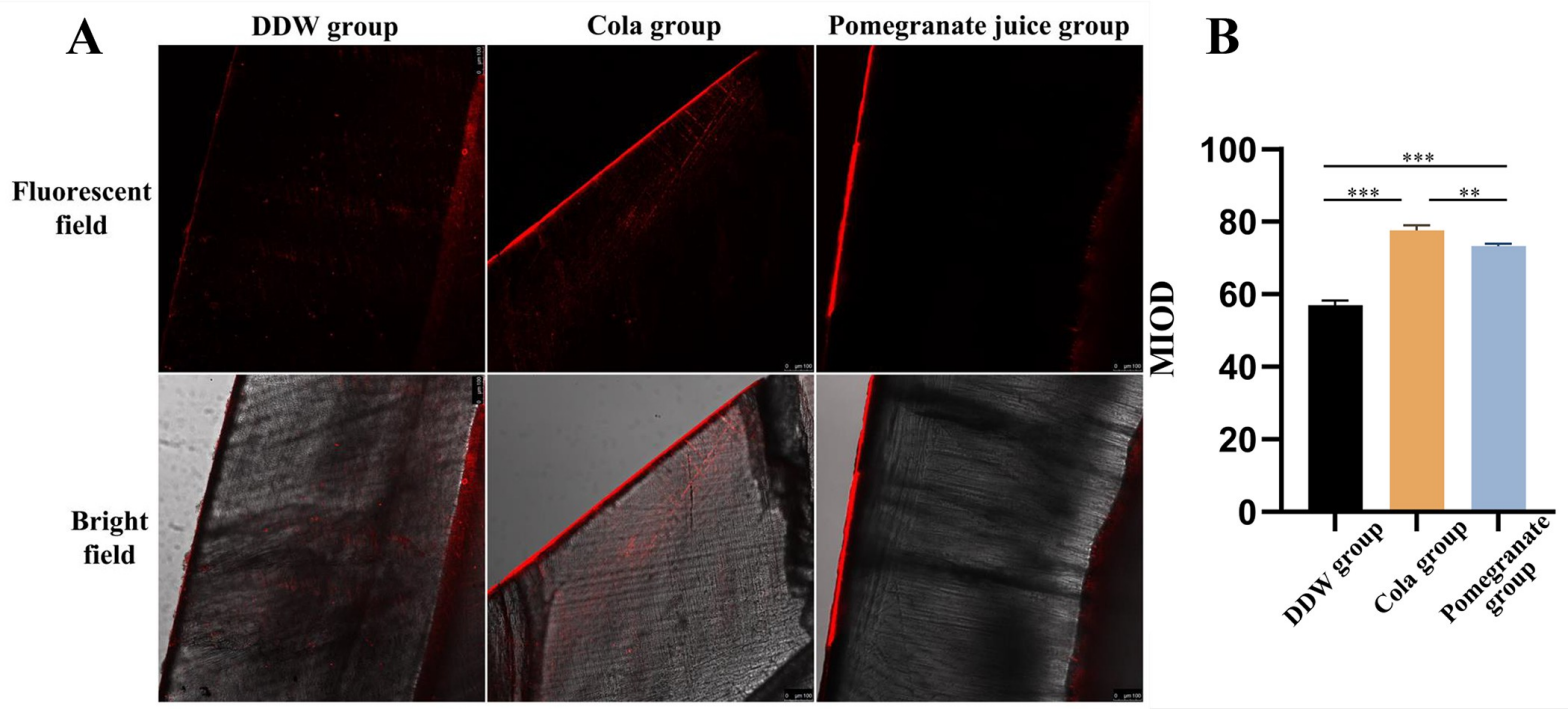
CLSM was used to detect the depth of demineralization in the enamel mass. (A) CLSM images of the specimens after rhodamine B staining, wherein the width of the red area reflects the demineralization depth in the enamel. (B) Comparison of the demineralization depths in the specimens from the deionized water, cola, and pomegranate juice groups. *P < 0.05, **P < 0.01, ***P < 0.001.

In Fig 4A, red areas in the CLSM images represent the depth of demineralization in the block, and black areas represent the unstained air and normal hard tissue. Only slight fluorescent dye penetration was observed in the deionized water group, whereas continuous uniform fluorescent bands were observed in the cola and pomegranate juice groups. Quantitative fluorescence analysis showed that the mean fluorescence intensities in the pomegranate juice and cola groups were significantly higher than that in the deionized water group. Furthermore, significant differences were observed between the deionized water group and the cola and pomegranate juice groups (Fig 4B).

### The pH, calcium, and phosphorus levels in the three experimental solutions

**Fig 5:**
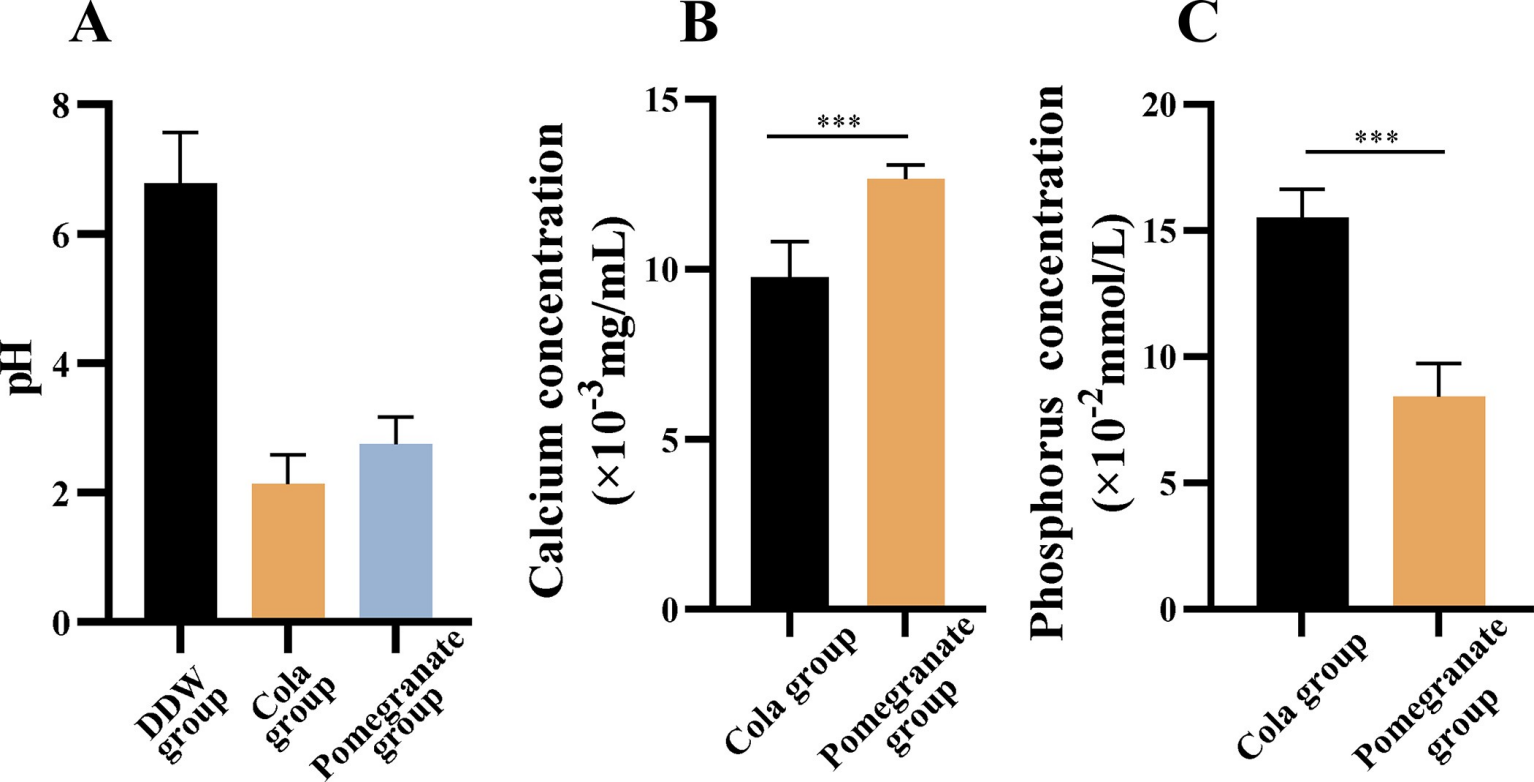
The pH, calcium, and phosphorus levels in the three solutions. (A) pH values of the three solutions; (B) Calcium concentrations in the solutions; (C) Concentration of phosphorus in the three solutions. *P < 0.05, **P < 0.01, ***P< 0.001.

As shown in Fig 5A, the pH of cola (2.32 ± 0.087) was lower than that of pomegranate juice (3.16 ± 0.16), whereas the pH of deionized water tended to be neutral at 6.78 ± 0.02. The calcium level in pomegranate juice (12.70±0.40) × 10^−3^ mg/mL was significantly higher than that of cola (*P* < 0.05; Fig 5B), whereas the concentration of phosphorus in cola (15.53±1.05) × 10^−2^ mmol/L was significantly greater than that in pomegranate juice (*P* < 0.05; Fig 5C).

## Discussion

Tooth erosion is defined as the pathological loss of dental hard tissue due to the chemical influence of acids [17]. The acids that cause tooth erosion are not inherent to the oral environment and can be divided into endogenous and exogenous, depending on the source. The most common source of exogenous acids is diet, such as acidic soft drinks, fruit juices, and the increasingly popular sports and energy drinks [18]. Pomegranate juice is becoming increasingly accepted by the general public due to its sweet taste and nutrient richness, but the effect of regular intake of this juice on teeth has not been reported yet [19]. The surface hardness of enamel can be used to assess the degree of demineralization. After demineralization for 14 days, the enamel hardness values in the cola and pomegranate juice groups were significantly lower than that in the deionized water group and statistically different from those at baseline, indicating that pomegranate juice causes significant demineralization of the enamel surface. In addition, the enamel demineralization exhibited a decreasing trend in speed from fast to slow over the 14-day period. Dissolution of the ions in the enamel alters its surface chemistry and decreases the solubility of the ions on the surface. With continued demineralization, placement of the block in pomegranate juice and cola can lead to the removal of more soluble material from the enamel surface; moreover, hydroxyapatite in the enamel is not re-precipitated with sufficient carbonate, leading to a decrease in the solubility of the ions in the [20].

The enamel is mainly composed of hydroxyapatite crystals, and the compounds in hydroxyapatite crystals, especially carbonates, are highly soluble in acids [21]. Enamel prisms are rod-like structures (cross-sectional diameter, 3–6 μm) formed by a group of similarly oriented hydroxyapatite crystals [22]. Enamel demineralization is categorized as type I (affecting the prism core), type II (affecting the prisms), and type III (presence of both types I and II demineralization) [23]. SEM showed that the enamel surface in the pomegranate juice group was rough, with a large number of pores; the enamel column was broken in large areas, the center of the enamel column was dissolved, and the porosity was greater than that observed in the cola and deionized water groups. In the deepest demineralized regions, some areas were shaped like craters of different sizes and shapes and partially connected into pieces, making it difficult to distinguish the boundary between normal and affected enamel. The blocks in the pomegranate juice group were thought to undergo type II demineralization. In the cola group, the enamel column was broken after demineralization. Some areas showed a honeycomb structure, and some were connected into pieces; consequently, and the boundaries between normal and affected enamel were distinguishable. Thus, it was speculated that the cola group underwent type III demineralization. The surface morphology of the blocks in the deionized water group was complete and uniform, and only small sporadic depressions were observed.

The type of acid further impacts the acid-etching potential of the juice by affecting the type of demineralization [24]. Pomegranate juice has an extremely high total phenolic content and antioxidant capacity and consists of various acids, mainly citric and malic acid [25]. Citric acid is present in water as a mixture of hydrogen ions, acid anions (citrate), and undissociated acid molecules; the hydrogen ions directly dissolve enamel by binding to carbonate or phosphate ions [26]. In addition to the action of hydrogen ions, citrate ions irreversibly chelate with calcium to form calcium citrate, accelerating calcium loss from the teeth [27]. Therefore, pomegranate juice causes demineralization of the enamel through the dual action of citric acid and is very harmful to the tooth surface. Notably, phosphoric acid, which is abundant in cola, has a stronger acid etching property than citric acid, which is abundant in pomegranate juice. On the one hand, phosphoric acid provides hydrogen ions at low pH, which directly dissolve enamel by binding to carbonate ions or phosphate ions; on the other hand, at a higher pH, the hydrogen ions in solution bind to calcium and dissolve enamel [28].

CLSM, widely used to assess the degree of enamel demineralization, combines visible light and laser to observe the penetration depth of the fluorescent dye and assess the degree of demineralization [29]. The cola and pomegranate juice groups showed continuous uniform fluorescent bands, whereas only mild fluorescent dye penetration was seen in the deionized water group in the current study. The mean fluorescence values in the cola (0.7761 ± 0.0111) and pomegranate juice (0.7332 ± 0.0047) groups were significantly greater than that in the deionized water group (0.5342 ± 0.0400), further confirming that pomegranate juice and cola can cause demineralization of the enamel surface.

In general, beverages with lower pH values have more potent acid etching effects; however, the pH of a beverage does not fully reflect its acid etching potential. Based on the role of ions in the demineralization process, differences in calcium and phosphate content in beverages can also affect the acid etching potential. The higher the calcium content in the juice, the slower the demineralization of the enamel [30]. Featherstone et al. confirmed that adding calcium to foods and beverages can prevent citric acid-induced enamel demineralization by binding to citrate and reducing the degree of demineralization [31]. In another study by Attin et al., the modification of acidic beverages with low calcium concentrations or a combination of calcium, phosphate, and fluoride resulted in protective effects against tooth erosion [32]. In the current study, the calcium content in pomegranate juice was markedly higher than that in cola. The dissolution of hydroxyapatite in blocks immersed in pomegranate juice was decreased in the presence of high amounts of calcium.

However, the experiments in this study were conducted in vitro and may not entirely simulate the oral environment. Thus, further in vivo studies are needed to verify the effect of pomegranate juice on the enamel surface morphology and surface hardness. In addition, the degree of acid etching by the juice is influenced by its temperature, the frequency of consumption, and the saliva [33–35]. Additional studies considering these factors are warranted in the future.

## Conclusions

Pomegranate juice has a sufficiently low pH to have a potential acid-etching potential on the enamel surface, leading to demineralization of the enamel surface, and long-term continuous consumption of pomegranate juice can lead to irreversible damage to tooth structure. Therefore, the publicity and education work should be strengthened in life to drink juice drinks reasonably and maintain good oral hygiene.

## Supporting information

S1 Data (ZIP)
